# How Target and Perceiver Gender Affect Impressions of HIV Risk

**DOI:** 10.3389/fpubh.2015.00223

**Published:** 2015-10-06

**Authors:** Alexander Barth, Ralf Schmälzle, Freda-Marie Hartung, Britta Renner, Harald T. Schupp

**Affiliations:** ^1^Department of Psychology, University of Konstanz, Konstanz, Germany

**Keywords:** risk perception, HIV, gender, trust, attractiveness

## Abstract

**Background:**

People do not use condoms consistently but instead rely on intuition to identify sexual partners high at risk for human immunodeficiency virus (HIV) infection. The present study examined gender differences of intuitive impressions about HIV risk.

**Methods:**

Male and female perceivers evaluated portraits of unacquainted male and female targets regarding their risk for HIV, trait characteristics (trust, responsibility, attractiveness, valence, arousal, and health), and willingness for interaction.

**Results:**

Male targets were perceived as more risky than female targets for both perceiver genders. Furthermore, male perceivers reported higher HIV risk perception for both male and female targets than female perceivers. Multiple regression indicated gender differences in the association between person characteristics and HIV risk. In male targets, only trustworthiness predicts HIV risk. In female targets, however, HIV risk is related to trustworthiness, attractiveness, health, valence (for male perceivers), and arousal (for female perceivers).

**Conclusion:**

The present findings characterize intuitive impressions of HIV risk and reveal differences according to both target and perceiver gender. Considering gender differences in intuitive judgments of HIV risk may help devise effective strategies by shifting the balance from feelings of risk toward a more rational mode of risk perception and the adoption of effective precautionary behaviors.

## Introduction

The human immunodeficiency virus (HIV) constitutes one of the world’s major risks to human health. Around 2.1 million people are estimated to be living with HIV in North America and Western and Central Europe (1). Despite increasing use of antiviral therapy, infection rates have remained virtually stable in these regions over the recent years. Furthermore, numerous campaigns have informed the public that unsafe sexual behavior is the primary way of contracting HIV (1, 2). However, while most people are well informed about safer sex practices and consistent condom use, various studies observed low perception of HIV risk and inconsistent and infrequent condom use in young adults (3–7). These findings suggest that when it comes to HIV or other sexually transmitted infections (STIs), knowing the facts is not sufficient to motivate consistent protective behavior.

Rather than relying consistently and reliably on safer sex practices, people appear to employ an array of strategies, such as “getting to know the partner” or “learning about his or her sexual history” to judge the likelihood to get infected with an STI (8, 9). Unfortunately, these prevention strategies are not effective but may induce a false sense of control over the risk. One particularly concerning finding is that people are prone to form immediate impressions about their risk for getting infected by a specific partner. Specifically, people who are infected with HIV often report that they were convinced that their partners were safe (10). Similarly, focus groups with college students revealed that these young adults often rely on their feelings about riskiness, i.e., they report that they “just know” whether a person is risky or safe – even when they do not know much about the respective person’s past sexual behavior or personality (6, 10–12). Thus, it appears that HIV risk perception is at least partly based on spontaneous impressions of others, and that “safe” impressions may undermine reliance on effective protection strategies.

Several recent studies support the notion that HIV risk perceptions are based on intuitive processes. With regard to self-report data, it has been repeatedly and consistently shown that participants can easily provide their impressions about the HIV risk of unacquainted opposite-sex individuals (13–15). In addition, when probed at the end of the experiment, participants could not explain how they arrived at their risk judgments and reported severe difficulties verbalizing “hunches” (15). Furthermore, a series of neuroscientific studies assessed key features of intuitive processes, such as speed of processing and affect. Event-related potential recordings (ERP) revealed that the brain responses to risky as compared to safe individuals diverged early in the processing stream (<300 ms), preceding systematic reasoning about health risks, and elicit a larger late positive potential, a specific ERP component that has been linked to affective evaluation processes (13–15). Furthermore, a functional neuroimaging study observed that larger HIV risk perceptions were associated with increased activation in regions of the saliency network, i.e., the anterior insulae and medial frontal cortex, which are also engaged by threatening and negative-affect related stimuli (16). Perhaps the strongest support for the intuitive nature of HIV risk perception comes from studies which showed the same photographs of persons in an implicit and explicit condition (16, 17). In the implicit condition participants did not evaluate HIV risk but performed only a simple memory task to ensure processing of the photographs. Categorizing data of this condition according to HIV risk ratings obtained in a subsequent explicit rating condition revealed similar ERP and fMRI correlates of risk processing for implicit and explicit conditions. Taken together, there is increasing evidence to support the notion that HIV risk perception is based on intuitive as opposed to analytic processing.

Because an STI or HIV infection does not lead to immediate health problems, there are no overt or observable signs that accurately indicate HIV or STI risk status. Accordingly, when people report that they “just know” the risk posed by a certain individual, impressions about the risk status are likely to be inferred from other personal characteristics. To address the issue, previous studies assessed the association of HIV risk with person trait characteristics, such as attractiveness, valence, health, trustworthiness, and responsibility. A main finding was that ratings of HIV risk, trust, and responsibility loaded on a common factor related to safeness in interpersonal relationship which was distinct from a “valence-approach” factor which had high loadings of valence, attractiveness, perceived healthiness, and the behavioral approach dimension “willingness to interact” (14). Previous research revealed that a low sense of responsibility and distrust was reliably named as a key feature characterizing persons with a high risk of HIV (18). Overall, there is growing evidence that the strategy to screen partners for their HIV risk may result from an intuitive, “gut-feeling” mode of risk perception related to the activation of a high at risk stereotype.

Undermining reliance on intuitive HIV risk judgments in favor of effective protection strategies may be a target for public health campaign. In order to devise effective strategies to educate intuitive processes (19), it seems relevant to determine whether there are gender differences in snap judgments about HIV risk. However, the issue of systematic gender differences has yet to be explored. Previous research usually focused on HIV risk perception of potential sexual partners, providing no systematic comparison of whether the gender of the perceiver or the target person results in systematic gender differences. Yet, reliable and consistent gender differences with regard to partner selection, resource distribution and trust in social life as well as the portrayal of HIV risk in the public have been reported (20, 21), raising the possibility for gender differences in HIV risk perception. One source for gender differences may lie in the portrayal of HIV risk in the public. For instance, public campaigns in the recent past often emphasized an increased risk in women due to an increased biological susceptibility for infection and gendered power dynamics (21). Alternatively, risk ratings may reflect infection rates, which are much higher for men as compared to women in Germany (22). A further source for gender differences regards systematic mean differences in perceived trustworthiness. Previous research showed that women are perceived as more trustworthy than men by male and female perceivers (23). According to the strong relationship of HIV risk and trustworthiness, one may accordingly posit gender differences when rating the HIV risk of men and women. Finally, one most important aspect of gender difference may not relate to systematic differences in mean ratings of HIV risk but rather concern differences in the kind of information associated with HIV risk. For instance, it is well established that attractiveness and health is more relevant for female than male partner selection, possibly reflecting the conjoint influence of evolutionary and socio-cultural factors (20). This raises the intriguing hypothesis of gender differences regarding the relationship of trait personality characteristics to HIV risk.

The main aim of the present study was to examine possible differences between the genders in the operation of snap judgments about HIV risk. To this end, perceivers (men and woman) were asked to spontaneously rate target pictures (male and female) regarding their risks for HIV, several trait characteristics (trust, responsibility, attractiveness, valence, arousal, and health) and willingness for interaction as proximal measure for approach or avoidance behavior. Regarding perceived HIV risk ratings, a first line of analysis examined mean differences of HIV risk as a function of Perceiver Gender and Target Gender. In a second stream of analysis, multiple regression analysis was conducted for the four groups (i.e., female perceiver/female target, male perceiver/female target, female perceiver/male target, and male perceiver/male target) to determine whether there are gender differences in the relationship of personality characteristics to HIV risk among genders.

## Materials and Methods

### Participants

Ninety-two volunteers, 49 female (53.26%), aged 18–28 years (*M* = 21.45, SD = 1.79), and 43 male (46.74%), aged 19–27 years (*M* = 21.72, SD = 1.96), were recruited on campus at the University of Konstanz. Eighty-nine participants (48 females) reported regarding themselves as heterosexual, one female and one male participant as homosexual, and one male participant regarded himself as bisexual at the time of data acquisition. Participants received either monetary reimbursement or course credits as compensation. Six participants had to be excluded from the analysis because they did not comply with the instructions, i.e., lack of variance in the data. Thus, the final sample comprised 46 (53.49%) female and 40 (46.51%) male participants. The study was conducted according to the guidelines of the Declaration of Helsinki and approved by the Ethic Review Board of the University of Konstanz. All participants provided written consent to the study protocol.

### Stimulus materials

The stimulus set consisted of colored photographs depicting 60 males and 60 females, which were retrieved with permission from a popular online photo-sharing community (www.flickr.com). To assure high ecological validity, stimuli showed a colored photo of a single person located in the foreground, with their face clearly visible. To be representative of the study’s target population in terms of age and race, only photographs of Caucasians between 18 and 35 years old were included. To resemble naturalistic viewing conditions and to facilitate impression formation, self-portraits exhibiting attire, socioeconomic status cues, and situational context features were included. Based on our previous research (13–16), stimuli were selected to represent a broad variation of HIV risk ratings. Specifically, by collapsing data from previous studies, the mean HIV risk rating was calculated for each image, sorted in ascending order, and, one picture from neighboring picture pairs was included in the stimulus set in order to select 60 out of 120 pictures. Each perceiver viewed the entire picture set showing same and opposite-sex persons (male and female target pictures) in random order.

### Stimulus ratings

Human immunodeficiency virus risk perception was assessed by asking participants “How likely do you think is it that this person is HIV-positive?” Healthiness was assessed with the question “How would you gage the state of health of this person?” For trustworthiness, participants stated “I find this person … [not at all trustworthy – very trustworthy]?” Responsibility was measured by asking participants “How would you gage the sense of responsibility of this person?” Valence was assessed by asking the question “Watching this person makes me feel … [very unpleasant – very pleasant?” Arousal was assessed by asking participants “Watching this person makes me feel … [very calm – very excited?” Attractiveness was assessed with the statement “I find this person … [very unattractive – very attractive]?” For willingness to interact participants should state “I … [don’t want to meet this person – do want to meet this person]?” All ratings were given on a 7-point scale, with greater numbers indicating that the respective characteristic is more pronounced.

### Procedure

The experiment was conducted in groups of 8–12 participants, who were seated at separate tables. After a general overview of the study and a brief questionnaire, instructions were provided and the seven ratings scales introduced. It was emphasized that there were no right or wrong answers. Each of the stimulus pictures was presented for 2 s, preceded by a 1 s fixation cross and a 1 s post-picture period after which perceivers were asked to evaluate the pictures on the rating scales, which included the rating question and a smaller version of the photograph. The order of the rating scales varied randomly from trial to trial. The investigator was present in the room throughout the experiment to assure proper conditions for data collection. Following the main experiment lasting ~40 min, participants filled out a brief post-experimental questionnaire, received reimbursement or course credits, were debriefed, and thanked.

### Statistical analyses

To determine whether the risk rating distributions of the four experimental groups defined by the factors “Target Gender” and “Perceiver Gender” show substantial variation in ascribed HIV risk, minimum, maximum, mean range, and variance of the risk ratings were calculated for each participant. Intra-class correlations (ICC, two-way random, mean) were calculated to determine inter-rater agreement for each of the groups comprising the 2 (Target Gender) and 2 (Perceiver Gender) combinations. Mean HIV risk ratings were analyzed using repeated measure analyses of variance with the within factor “Target Gender” (male vs. female) and the between factor “Perceiver Gender” (men vs. women). The relationship between HIV risk ratings and other trait person characteristics was examined by calculating Pearson correlation coefficient, multiple regression, and mediation analysis.

## Results

### HIV risk rating distribution

**Figure 1:**
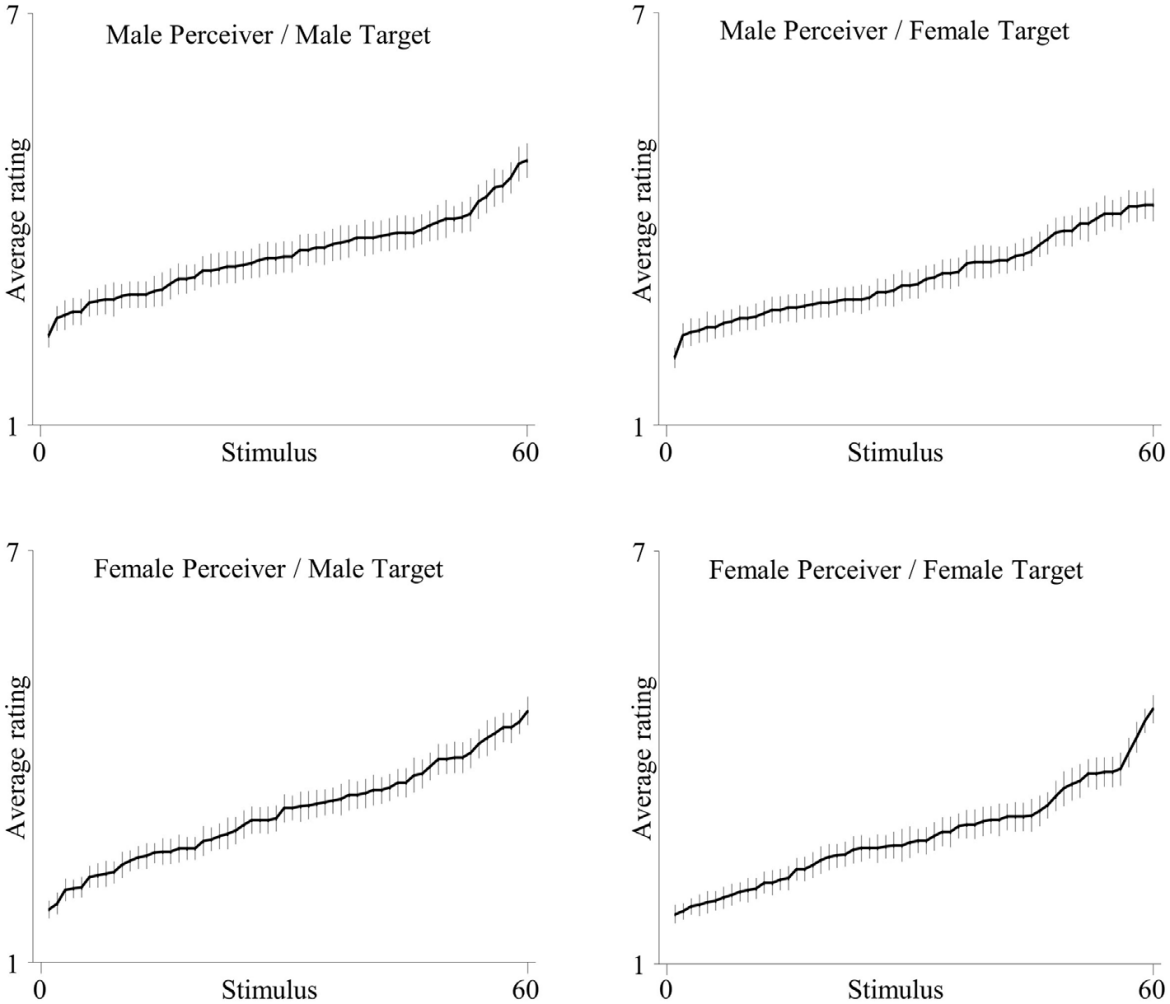
**Mean HIV ratings rank-ordered for the four groups defined by the factors “Target Gender” and “Perceiver Gender**.”

To examine gender differences it is necessary to demonstrate that the four groups defined by the factors of “Perceiver Gender” and “Target Gender” show substantial variation in ascribed HIV risk. As shown in Figure 1, in each group mean HIV risk ratings increased from very low (minimum = 1.26) to very high (maximum = 5.84). Furthermore, participants in all four groups used the full range of the scale (*x* = 5.0) and showed substantial variance (*s*^2^ = 1.76) in perceived HIV risk. Overall, providing the grounds to examine gender differences, these analyses demonstrate that perceived HIV risk showed substantial variance for female and male target pictures as well as female and male perceivers.

### Inter-rater agreement

Table 1 illustrates the inter-rater agreement for HIV risk perception and trait personality characteristics. For all groups and all measures, ICC coefficients were very good (ICC > 0.75).

**Table 1:** **Intra-class correlation coefficients for HIV risk perception and trait personality characteristics, separately for the four groups defined by the factors “Target Gender” and “Perceiver Gender**.”

	Male targets		Female targets	
	Male perceivers	Female perceivers	Male perceivers	Female perceivers
HIV risk	0.88	0.94	0.91	0.94
Trustworthiness	0.94	0.97	0.94	0.97
Health	0.95	0.97	0.95	0.96
Valence	0.92	0.96	0.92	0.95
Arousal	0.75	0.85	0.86	0.85
Attractiveness	0.96	0.98	0.97	0.97
Interaction	0.92	0.96	0.94	0.95
Responsibility	0.94	0.97	0.93	0.97

### HIV risk perception

A significant main effect of “Target Gender” was observed, *F*(1,118) = 15.23, *p* < 0.01, η^2^ = 0.11, which indicated that male targets were perceived as more risky than female targets (see Table 2). Furthermore, a significant main effect of “Perceiver Gender” was observed, *F*(1,118) = 17.5, *p* < 0.01, η^2^ = 0.13, indicating that male perceivers provided higher HIV risk ratings as compared to female perceivers. However, the two factors did not interact, Target Gender × Perceiver Gender, *F*(1,118) = 0.003, *p* = 0.96, η^2^ < 0.01.

**Table 2:** **Mean HIV risk ratings and SDs for the four groups defined by the factors “Target Gender” and “Perceiver Gender**.”

	Male targets (*N* = 60)		Female targets (*N* = 60)		Male ≠ female	All targets (*N* = 120)	
	*M*	SD	*M*	SD		*M*	SD
Male perceivers (*N* = 40)	3.48	1.3	3.15	1.2	*t* = 6.24, *p* < 0.01	3.32	1.25
Female perceivers (*N* = 46)	3.17	1.27	2.85	1.24	*t* = 7.00, *p* < 0.01	3.01	1.25
Male ≠ female	*t* = 2.16, *p* = 0.04		*t* = 2.11, *p* = 0.04			*t* = 2.58, *p* = 0.01	
All perceivers (*N* = 86)	3.32	1.28	2.99	1.22	*t* = 9.42, *p* < 0.01	3.16	1.25

### Association of perceived HIV risk and other trait person characteristics

In order to determine the association of perceived HIV risk with trait person characteristics, correlations coefficients were calculated separately for all of the four groups, i.e., female perceiver/female target, male perceiver/female target, female perceiver/male target, and male perceiver/male target. As shown in Table 3, there was a robust and consistent relationship between perceived HIV risk and perceived trustworthiness and responsibility in each of the four groups (*r*’s > 0.85, *p* < 0.001). While the profile of correlations across the four groups showed overall similarities, there were, however, also group differences. Specifically, correlations of perceived HIV risk and perceived attractiveness, valence, arousal, health, and willingness for interaction varied between the groups.

**Table 3:** **Correlations between mean ratings of the seven trait dimensions with HIV risk ratings for the respective rating groups**.

	Male targets		Female targets	
	Male perceivers	Female perceivers	Male perceivers	Female perceivers
Trustworthiness	−0.86**	−0.89**	−0.88**	−0.92**
Health	−0.56**	−0.64**	−0.65**	−0.77**
Valence	−0.69**	−0.71**	−0.45**	−0.80**
Arousal	0.66**	0.60**	0.47**	0.87**
Attractiveness	−0.42**	−0.43**	−0.13	−0.35**
Interaction	−0.66**	−0.60**	−0.30*	−0.72**
Responsibility	−0.85**	−0.91**	−0.87**	−0.91**

*p ≤ 0.05.

**p ≤ 0.01.

To determine whether there are reliable differences between the groups regarding the association of perceived HIV risk with trait person characteristics, multiple regression models were calculated separately for each group. As shown in Table 4, the results indicated target-related differences primarily with respect of target gender. For male targets, trustworthiness was the only significant predictor in the male and female perceiver group, β_male_perceivers_ = 0.75, *p* = 0.02; β_female_perceivers_ = 0.89, *p* = 0.01. For female targets, perceived HIV risk was also significantly predicted by trustworthiness. In addition, several further person characteristics emerged, which, however, varied between male and female perceiver groups. For male perceivers, perceived HIV risk was predicted significantly by health, β_male_perceivers_ = 0.38, *p* < 0.01, valence, β_male_perceivers_ = 0.78, *p* < 0.01, attractiveness, β_male_perceivers_ = −0.63, *p* = 0.01, and trustworthiness, β_male_perceivers_ = 0.54, *p* = 0.02. For female perceivers, perceived HIV risk was predicted significantly by health, β_female_perceivers_ = 0.41, *p* < 0.01, arousal, β_female_perceivers_ = 0.27, *p* = 0.01, attractiveness, β_female_perceivers_ = −0.34, *p* < 0.01, and also trustworthiness, β_female_perceivers_ = 0.77, *p* = 0.01.

**Table 4:** **Predictors of perceived HIV risk for the four groups defined by the factors “Target Gender” and “Perceiver Gender**.”

	Male perceivers				Female perceivers			
	B	SE	β	*t*	B	SE	β	*t*
**MALE TARGETS**								
Trustworthiness	−0.525	0.226	−0.752*	−2.324	−0.639	0.242	−0.886*	0.242
Health	−0.181	0.120	−0.265	−1.508	−0.120	0.100	−0.161	0.100
Valence	0.481	0.282	0.525	1.707	0.118	0.235	0.139	0.235
Arousal	0.352	0.197	0.230	1.789	−0.250	0.184	−0.148	0.184
Attractiveness	0.044	0.125	0.068	0.349	0.039	0.125	0.069	0.125
Interaction	−0.156	0.235	−0.201	−0.665	0.176	0.219	0.253	0.219
Responsibility	−0.077	0.193	−0.106	−0.401	−0.276	0.174	−0.362	0.174
*R*^2^	0.772				0.849			
**FEMALE TARGETS**								
Trustworthiness	−0.463	0.186	−0.543*	−2.497	−0.561	0.222	−0.766*	−2.534
Health	−0.286	0.095	−0.378**	−3.017	−0.333	0.071	−0.414**	−4.674
Valence	−0.709	0.233	−0.783**	−3.044	0.292	0.222	0.307	1.315
Arousal	−0.086	0.152	−0.074	−0.565	0.462	0.158	0.274**	2.928
Attractiveness	0.362	0.137	0.628*	2.634	0.247	0.082	0.335**	3.021
Interaction	0.306	0.192	0.449	1.590	−0.154	0.186	−0.186	−0.830
Responsibility	−0.030	0.188	−0.035	−0.158	0.093	0.142	0.121	0.658
*R*^2^	0.855				0.920			

*p ≤ 0.05.

**p ≤ 0.01.

To explore the relationship between HIV risk perception, personality trait characteristics, and a proximal variable for behavior, i.e., willingness to interact, an exploratory mediational model was specified with HIV risk perception as mediator, the six trait personality characteristics as predictors and willingness to interact as dependent variable. Significant direct effects were observed for trustworthiness (β = 0.3, *p* < 0.001), valence (β = 0.65; *p* < 0.001), attractiveness (β = 0.26; *p* < 0.001), and arousal (β = 0.10; *p* < 0.001). To test the amount of mediation, indirect effects were estimated using a non-parametric bootstrap approach (*N* = 2000). Significant indirect effects were observed for trustworthiness (β = −0.08; 95% CI = −0.13 to −0.02), health (β = −0.03; 95% CI = −0.05 to −0.01), and attractiveness (β = 0.04; 95% CI = 0.01–0.06).

## Discussion

The present study examined perceptions of HIV risk with a focus on the gender of targets and perceivers. Perceived HIV risk was increased for male targets and male perceivers. Furthermore, the association of perceived HIV risk and trait person characteristics differed for male and female target pictures. For male targets, HIV risk was only associated with ratings of trustworthiness, in multiple regression analysis, and no other personality characteristic made an independent, further contribution. For female targets, in addition to trustworthiness, attractiveness, health, valence (male perceivers), and arousal (female perceivers) were predictive. Thus, first impressions about HIV risk for female and male target pictures were based on different sources of information. These gender differences may have implications for the design of HIV-prevention campaigns. Such campaigns often convey high-risk stereotype information, which may contribute to the associative structure of personality characteristics underlying first impressions of HIV risk.

A key question raised by the present data is how to account for the gender-differentiated face of HIV risk perception. While a cogent explanatory framework is missing, evolutionary and socio-cultural considerations of (a) differences between the genders related to partner selection and resource distribution and trust in social life (24), and (b) the portrayal of HIV risk in the public (21) may provide at least partial explanations of the observed effects. Specifically, gender differences related to partner selection have been well documented in cross-cultural studies (20). Physical attractiveness and health are more important for men selecting partners, while women consider social status and financial resources more important (25). These gender differences in person characteristics related to partner selection may be reflected in the association of perceived HIV risk and trait person characteristics. Specifically, attractiveness and health were only significant contributors in predicting HIV risk in female targets and were not observed for the male target pictures. Interestingly, these gender differences in target picture evaluation were similarly observed for female and male perceivers. Thus, rather than being related to partner selection, the associations between HIV risk and trait person characteristics seem to be shared between female and male perceivers. The present data also help to clarify the relationship of perceived attractiveness and perceived HIV risk. Neither the hypothesis that attractiveness increases HIV risk (i.e., higher likelihood of many partners) nor the “what is beautiful is good”-heuristic has received strong support in previous research (15, 26–28). While correlation analysis has suggested a moderate relationship between attractiveness and perceived HIV risk, this analysis does not account for shared variance among related constructs, such as health and valence. In contrast, when using multiple regression analysis to identify predictors of HIV risk, attractiveness and health conjointly contribute to HIV risk, but only in female targets. While multiple regression analysis is well-suited to identifying variables that significantly contribute to the prediction of HIV risk, due to multicollinearity, exact interpretation of standardized beta-weights is limited (29). By using stimuli in which relevant trait characteristics are systematically varied, future research may clarify this issue.

A somewhat different perspective on the present findings arises from considering how HIV and AIDS are portrayed in public media. Especially in the early phase of the disease’s history, HIV and AIDS have always been strongly connected with sexual intercourse between males, and were even referred to as “the gay plague.” Until today, public discussion in western countries is still dominated by men-related topics, like debates about the usefulness of pre-exposure prophylaxis for gay men (30). The extensive and continued consideration of HIV risk factors for homosexual and heterosexual men may accordingly have contributed to a clear picture of men’s HIV risk (i.e., male targets) as being centered on variables of trustworthiness and responsibility. Conversely, women did not initially appear in public perception and discussion of HIV (31, 32), and for almost a decade, women did not even take part in neuropsychological studies on HIV (33). Only recently have women received more attention (34); however, this view is restricted to women from the global South and does not extend to women in Western countries. Furthermore, the “vulnerability paradigm” added a further imbalance to the gender-related perception of HIV (21). Specifically, women are often referred to as having less sexual autonomy and being more vulnerable while men are seen as active transmitters of the disease (35). Thus, women appear to be in need of protection while men appear to pose a potential threat. Accordingly, the nuanced multi-faceted association of personality characteristics and perceived HIV risk in women (i.e., female targets) may be the result of social-cultural factors related to sexual autonomy and social status as well as a less developed high HIV risk stereotype. Overall, these hypotheses regarding the mechanisms of gender differences are rather speculative and need to be examined in future research including the consideration of gender roles and social norms across cultures.

A further main finding of the present study was that HIV risk ratings differed for genders. Male target images received higher risk ratings compared to female images, and the effect was similarly observed for male and female perceivers. The finding may reflect knowledge, i.e., 4:1 infection ratio of men/woman in Germany (22) or the view that men are the active transmitters of the disease. In addition, male and female raters differed significantly in their risk ratings, with male raters providing higher risk ratings than female raters. This finding somehow contrasts with previous research showing that woman felt more at risk than men when asked about their personal feelings of safety (36). These conflicting findings may be reconciled by differentiating between perceived risk in another person, i.e., other-person risk and feelings of vulnerability for the self (37). Furthermore, while the interaction among the factors “Perceiver Gender” and “Target Gender” was not significant, it is noteworthy that the respective group of potential partners for the predominantly heterosexual sample received rather similar risk ratings.

Research on the mechanisms of first impressions of HIV risk can provide key insights into the conflict between “risk-as-analysis” and “risk-as-intuition.” Specifically, considering the characteristics of intuition can explain the paradox that people may “know” how to protect themselves but still occasionally refrain from safer sexual practices because they “feel” that the current partner does not pose a risk. Such intuitions often occur quickly and effortlessly, without conscious awareness, and the resulting impressions seem self-evidently valid (19). Along with additional situational constraints, i.e., shame and embarrassment when discussing HIV testing or protection (38), dislike and negative attitudes toward condoms (4, 5, 39), and the heat of the moment (40), the impression of safety may induce a false sense of control and risk protection (41). Furthermore, it seems that there are no preconditions for acquiring first impressions of HIV risk. The low base rate of HIV, the under-representative sampling, and the lack of corrective feedback strongly question reliance on intuition in this domain. Thus, unlike many other areas of social life in which first impressions may appear highly valid (42), intuitive HIV risk perception is fallible and provides an illusory control of risk. This line of reasoning might add to current approaches for HIV prevention. Challenging intuitive HIV risk perceptions may be considered as a strategy for promoting safer sex practices. In this regard, a study by Thompson et al. (41) is promising. Specifically, condom use increased in an intervention group, in which participants were reminded about failures to rely on safe sexual practices and directly experienced their inability to judge HIV-status based on visual appearance. Thus, the direct experience of the fallibility of the intuitive judgment of HIV risk may provide a further tool for HIV prevention.

In a similar vein, findings of gender differences in HIV risk perception could be informative for designing interventions that reduce HIV risk behavior. While male compared to female targets received higher HIV risk ratings from both genders, female participants provided systematically lower HIV risk ratings than males. One possible explanation for this effect is that mass media HIV campaigns in Central and Western Europe focus on high-risk groups. As a consequence, these types of campaigns may have fostered an implicit HIV risk stereotype socially distant to the well-educated and high social status college woman participating in the present study. Thus, revealing the gender differences associated with HIV risk perception can provide a foundation for the tailoring of HIV campaigns to respective subgroups based on gender and social status. This perspective is in line with recent suggestions to promote gender and culture sensitive mass media campaigns (43, 44).

Finally, several caveats need to be acknowledged. Specifically, the finding that male and female participants can easily provide perceptions of HIV risk for male and female targets’ does not imply that participants rely on the illusory control strategy to screen their partners for HIV risk. While there is reason to assume that reliance on an intuitive mode of risk perception is increased in a “hot” context of dating (45), future studies need to determine the factors increasing the reliance on intuition considering sexual experience and STI history as moderating factors. In addition, while ICC coefficients demonstrate high inter-rater agreement for the self-report measures collected in the present study, a possible limitation is the measurement of HIV risk and personality characteristics by a single-item measure. A further limitation of the present study regards the relationship between perceived HIV risk and protective sexual behaviors (46). For exploratory purposes, a mediation model has been specified in which trait personality characteristics were used to predict willingness to interact either directly or indirectly via HIV risk perception. In addition to direct effects for trustworthiness, valence, attractiveness, and arousal, results also revealed significant indirect effects for trustworthiness, health, and attractiveness. While these findings are consistent with the hypothesis of a mediating role HIV risk perception on willingness to interact, cross-sectional analysis is limited in revealing causal relationships. Accordingly, prospective studies are needed to examine the relationship between perceived HIV risk and protective sexual behaviors.

## Conclusion

The present research examined gender differences in first impressions of HIV risk. The findings revealed gender differences in intuitive impressions of HIV risk. Analyzing judgments of HIV risk and trait person characteristics for same and opposite-sex persons revealed increased risk perceptions for male perceivers as well as male targets and systematic gender differences in the structure of person characteristics associated with HIV risk. While trustworthiness was the only variable predictive of perceived HIV risk in male target images, a more refined pattern emerged for female targets, where HIV risk was predicted by trustworthiness as well as attractiveness and health. Considering these gender differences may help to optimize prevention strategies that prevent dangerous intuition-based strategies (8) and promote more deliberate strategies of risk perception and precautionary behaviors.

## Conflict of Interest Statement

The authors declare that the research was conducted in the absence of any commercial or financial relationships that could be construed as a potential conflict of interest.
